# Supplementary material for: MetaRibo-Seq measures translation in microbiomes
Source: Nat Commun. 2020 Jun 29;11:3268. doi: 10.1038/s41467-020-17081-z (PMC7324362; doi:10.1038/s41467-020-17081-z)
Supplement: Supplementary file 10 — Supplementary Data 7 [file 41467_2020_17081_MOESM10_ESM.zip › File2/Confidence_High_Taxonomy/126883_out.krona.html]

Javascript must be enabled to view this page.

members
magnitude
magnitudeUnassigned
count
unassigned
taxon
rank

126883\_out

2

superkingdom
2
2

phylum
2
1239

class
186801
2

order
2
186802

186803
1
family


SRS142527\_contig\_number\_9112
1410626
1
species

family
541000
1

1
216851
genus


SRS893187\_contig\_number\_16254
1
853
species
